# Early Activation of the Innate Immunity and Specific Cellular Immune Pathways after Vaccination with a Live Intranasal Viral Vaccine and Challenge with Bovine Parainfluenza Type 3 Virus

**DOI:** 10.3390/vaccines10010104

**Published:** 2022-01-11

**Authors:** Piet Nuijten, Natalie Cleton, Jeroen van der Loop, Birgit Makoschey, Wilco Pulskens, Geert Vertenten

**Affiliations:** MSD Animal Health, 5831 AN Boxmeer, The Netherlands; piet.nuijten@merck.com (P.N.); nataliecleton@gmail.com (N.C.); jeroen.vanderloop@merck.com (J.v.d.L.); birgit.makoschey@merck.com (B.M.); wilco.pulskens@merck.com (W.P.)

**Keywords:** intranasal vaccination, innate immunity, specific immunity, gene expression

## Abstract

Bovine parainfluenza type 3 (BPIV3) and bovine respiratory syncytial virus (BRSV) may cause bovine respiratory disease (BRD) in very young calves, and therefore vaccination should induce protection at the youngest age and as quickly as possible. This can be achieved by intranasal vaccination with a vaccine containing live attenuated BRSV and BPIV3 virus strains. The objective of this study was to measure gene expression levels by means of RT-qPCR of proteins involved in the innate and adaptive immune response in the nasopharyngeal mucosae after administration of the above-mentioned vaccine and after challenge with BPIV3. Gene expression profiles were different between (i) vaccinated, (ii) nonvaccinated-challenged, and (iii) vaccinated-challenged animals. In nonvaccinated-challenged animals, expression of genes involved in development of disease symptoms and pathology were increased, however, this was not the case after vaccination. Moreover, gene expression patterns of vaccinated animals reflected induction of the antiviral and innate immune pathways as well as an initial Th1 (cytotoxic) cellular response. After challenge with BPIV3, the vaccinated animals were protected against nasal shedding of the challenge virus and clinical symptoms, and in parallel the expression levels of the investigated genes had returned to values that were found before vaccination. In conclusion, in comparison to the virulent wild-type field isolates, the two virus strains in the vaccine have lost their capacity to evade the immune response, resulting in the induction of an antiviral state followed by a very early activation of innate immune and antiviral responses as well as induction of specific cellular immune pathways, resulting in protection. The exact changes in the genomes of these vaccine strains leading to attenuation have not been identified. These data represent the real-life situation and can serve as a basis for further detailed research. This is the first report describing the effects on immune gene expression profiles in the nasal mucosae induced by intranasal vaccination with a bivalent, live BRSV-BPI3V vaccine formulation in comparison to wild-type infection with a virulent BPI3V strain.

## 1. Introduction

Bovine parainfluenza type 3 (BPIV3) and bovine respiratory syncytial virus (BRSV) are single-stranded RNA viruses and members of the families *Pneumoviridae* and *Paramyxoviridae*, respectively. BRSV is the species-specific family member of human respiratory syncytial virus that causes serious morbidity and mortality in very young children. BRSV infection of calves has been considered as an experimental model for HRSV [1]. Both bovine viruses; BRSV and BPIV3, may cause bovine respiratory disease (BRD), which is a complex disease and can involve multiple viruses and bacteria. The disease symptoms can vary from subclinical, mild cough and fever, to severe pulmonary disease and death.BRD often develops in calves during the first weeks of life, facilitated by immunosuppressive circumstances and stressors such as transportation, mixing with other calves, and suboptimal environmental conditions [2,3].

Since BRD is one of the main diseases in young calves and has a large impact on animal welfare and performance, as youngstock but also later in life [4,5,6], vaccines have been developed to protect these young animals against infection in their first weeks of life. To obtain protection as young as possible, a vaccine must be suitable to be administered at a very young age and provide a quick onset of immunity [7,8]. However, the immune system of the calf is not completely matured in early life and may not respond appropriately to certain vaccines [9,10].

Protection against clinical symptoms and nasal shedding caused by infections with BRSV and BPIV3 viruses has been achieved by vaccination with an intranasal vaccine containing a BRSV and a BPIV3 attenuated live virus strain (Bovilis^®^ INtranasal RSP^TM^ Live, MSD Animal Health, Boxmeer, The Netherlands). This vaccine has demonstrated an early onset of protection [11]; five days for BRSV and seven for BPIV3, which might be the combined result of the mucosal innate response and early activation of the acquired immune system. The immune response against BRSV has been the subject of many studies because of its importance as a model for human RSV infections, and was reviewed recently [1,12,13], but the early mucosal immune response has never been investigated after intranasal administration with live attenuated vaccine strains, nor the response after wild-type BPIV3 infection or BPIV3 challenge after vaccination. Therefore, we aimed to measure gene expression of proteins involved in the innate and adaptive immune response in the nasopharyngeal mucosae after vaccination with the live vaccine and after challenge with BPIV3.

The data described here were collected during a study that was performed to determine the onset of immunity in order to license Bovilis^®^ INtranasal RSP^TM^ Live in Europe. Besides the samples and observations to determine safety and efficacy parameters, additional sampling and analyses were included for the objective of this immunological investigation. This may reveal the immunological mode of action leading to this early onset of protection, which will provide a better understanding of the immunological characteristics of a decreased virulence of potential vaccine strains and their ability to induce immunity.

## 2. Materials and Methods

### 2.1. Study Design

This study was performed as part of the required onset-of-immunity study to license Bovilis^®^ INtranasal RSP^TM^ Live in Europe (https://mri.cts-mrp.eu/Veterinary/Downloads/NL_V_0257_001_PAR.pdf, accessed on 1 November 2019) and conducted under European and Dutch law for animal experiments and welfare (approved by the national Animal Experiments Committee under the Animal Experimental Plan BCL14.034).

Besides the sampling and observation to determine safety and efficacy parameters, additional sampling and analyses were included for the objective of this immunological investigation. Thirteen colostrum-deprived calves, free of maternally derived antibodies (MDA) against BRSV and BPIV3, were divided into two experimental groups; one group of six calves was vaccinated intranasally and one group of seven calves received no vaccination. Seven days later both groups were challenged with a BPIV3 field strain by aerosol and efficacy was measured by investigating nasal shedding of BPIV3 and clinical signs, including rectal temperatures, during the two weeks after challenge. Animals were observed for general health during the entirety of the study.

Nasal brushes were used to take samples of the nasopharyngeal mucosae on days 0, 2, and 5 (vaccinated group) and on days 7, 9 and 12 (both groups). The samples of the nasal brushes were immediately fixed, mRNA was extracted, and qPCR was performed using an RT2 Profiler PCR array by QIAGEN GmbH (Hilden, Germany) for 84 immunity pathway target genes (see https://www.qiagen.com/us/shop/new-products/rt2-profiler-pcr-arrays/, accessed on 1 December 2018). Resulting Ct values, representing gene expression levels, were analyzed after normalization by comparing fold-changes in gene targets between paired samples from individual animals at different time points.

### 2.2. Animal Study

New-born colostrum-deprived calves from Dutch commercial breeders were randomly assigned to a treatment group based on time of arrival. The calves were housed at the animal facilities under BSL-2 containment. Calves were given milk replacer from the first day of arrival and were also fed calf starter from approximately 10 days of age. Water was available ad libitum. To prevent bacterial infections, calves were given 10 g paromomycin sulphate with the milk replacer twice daily until 10 treatments had been given, and from day of arrival 75 mg ceftiofur was injected subcutaneously daily for a minimum of five days. Antibiotic treatment is not expected to interfere with intranasal vaccination with live viruses. The calves were observed daily for general health. The animals in the vaccinated group were four to eight days of age at the time of vaccination and colostrum deprived. They had an acclimatization period of at least four days. The freeze-dried vaccine (Bovilis^®^ INtranasal RSP^TM^ Live) was dissolved in the diluent and 2 mL was administered intranasally (1 mL per nostril) using a syringe without a spraying device.

The heterologous BPIV3 challenge virus was harvested from a calf suffering from BPIV3 infection and passaged once in bovine embryo lung cells. A target titer of 6.7 log_10_ TCID_50_ BPIV3 in 10 mL challenge inoculum was used per calf, using a nebulizer (Tyco Healthcare, Neustadt a.d. Donau, Germany). The calves were restrained, and a face mask connected to the nebulizer by a tube was placed over nose and mouth, making sure that the animal had to breath in the mask. Clinical signs specific for respiratory disease (nasal discharge, respiratory rate and coughing; scoring according to McGuirk [14]) and body temperature were monitored from two days before challenge until the end of the experiment. To follow virus shedding, nasal swab samples (Dryswab MW102, MWE, Wiltshire, UK) were taken daily and stored in cold sterile transport medium (Sigma Virocult MW950, MWE, Wiltshire, UK) for virus titration on bovine embryonic lung (BEL) cells. To collect a nasopharyngeal mucosal sample containing mainly epithelial and white blood cells for PCR, nasal brushes (Dryswab™ MW126, MWE, Wiltshire, UK, veterinary nasopharyngeal with nylon brush for large animals) were used. The nasal brush samples were taken from alternating nostrils on sequential time points and the tip of the brush was immediately placed in 3 mL RNAlater solution (Invitrogen, ThermoFisher Scientific, Waltham, MA, USA).

### 2.3. Virus Titration

The BPIV3 titers of the nasal swabs were determined by titration followed by an immunoperoxidase monolayer assay staining. Five-fold serial dilutions were made in micro-titer plates seeded with a suspension of BEL cells. After an incubation period of 5–6 days at 37 °C and 5% CO_2_ in a humidified atmosphere, the plates were fixated by formalin solution and an immune-peroxidase-monolayer-assay (IPMA) was performed for BPIV3 detection using a horseradish-peroxidase-labelled anti-BPIV3 conjugate and a peroxidase substrate kit. After staining, wells were determined positive or negative for BPIV3 virus based on visual presence of staining. The titer was calculated by the method of Spearman and Kaerber and expressed in log_10_ TCID_50_/mL.

### 2.4. Virus Neutralization Serum Antibody Titers

A virus neutralization assay was used to test complement-inactivated serum samples for the presence of neutralizing antibodies. Serial two-fold serum dilutions were prepared in micro titer plates and mixed with an equal volume containing a standard amount of BPIV3. After the serum-virus pre-incubation period, Bovine Embryo Lung cells were added. Then cells were cultivated for 5–7 days at 37 °C and 5% CO_2_ in a humidified atmosphere and then examined for the presence or absence of BPIV3 by performing an IPMA as described above. The titer of the sample was calculated as the mean log_2_ value of the reciprocal of the highest (serum) dilution where no virus infection was demonstrated. Titers of 2.0 and lower are considered negative since in some cases low background signal causes a non-specific signal.

### 2.5. RT-qPCR Assay

The nasopharyngeal mucosal brush material was kept at 4 °C for at least 24 h after sampling to be appropriately fixed. One aliquot of 500 µL concentrated cell solution was used to isolate mRNA and perform a qPCR. RNA was isolated using a modified QIAGEN (Hilden, Germany) RNeasy Micro Kit (Cat.No. 74004) according to the manufacturer’s instructions. The experimental RNA samples were converted into strand cDNA using the QIAGEN RT2 First Strand Kit. Then the cDNA templates were mixed with ready-to-use RT2 qPCR Master Mixes for the RT2 Profiler PCR (QIAGEN, Hilden, Germany) and aliquoted into each well of the same plate containing pre-dispensed gene specific primer sets. Results were expressed as Ct values and exported to Excel for data analysis.

### 2.6. Statistical Analyses Nasal Shedding of BPI3V and Clinical Scores

For statistical analysis of nasal shedding, the sum of the clinical scores and clinical symptoms over time, an ANOVA or a corresponding nonparametric test (Kruskal Wallis) was used. If a result was significant, the Dunn’s post hoc test was applied to compare the groups. Tests were two-sided at a level of significance of 0.05.

### 2.7. Data Analysis RT-qPCR

Ct values <30 were considered as true positive signals. Samples with Ct values of 30–35 were flagged as ‘ambiguous’ because of the limit of quantification. Due to the relatively low levels of target-gene RNAs with Ct values between 30 and 35, the actual fold-change may have been greater than the calculated fold-change, but they were still included in analysis. Ct values of >35 were considered below the detection level of the qPCR and were flagged during analysis as ‘actual fold-changes cannot be calculated’.

A set of five reference genes was included for each sample needed for correction based on general transcription activity. Each target gene per sample was corrected by the average of the five reference genes and transformed according to the formula (2^−(Ct-value target gene − Average Ct value reference genes)^) to create a normal distribution for statistical analysis [15]. These corrected values were used to calculate the change in the target gene expression between two samples taken at two points in time from the same animal. This was expressed as the fold-change between the two time points for a specific target gene. Arbitrary criteria were set to indicate a biologically significant change in expression level: a change of more than 2-fold combined with *p*-values less than 0.01 and a change of more than 4-fold combined with *p*-values less than 0.05. The *p*-values were calculated using a Student’s *t*-test (two-tail distribution with paired samples) on the average corrected values between two time points within each test group (Supplemental File S1).

## 3. Results

### 3.1. Efficacy Parameters

Animals were MDA-negative at the start of the study and at the time of challenge were still seronegative (Table 1). Animals only seroconverted 14 days after challenge and average group titers went up to 7.3 log2 in the vaccinated-challenged group and up to 4.9 log2 in the nonvaccinated-challenged group.

**Figure 1:**
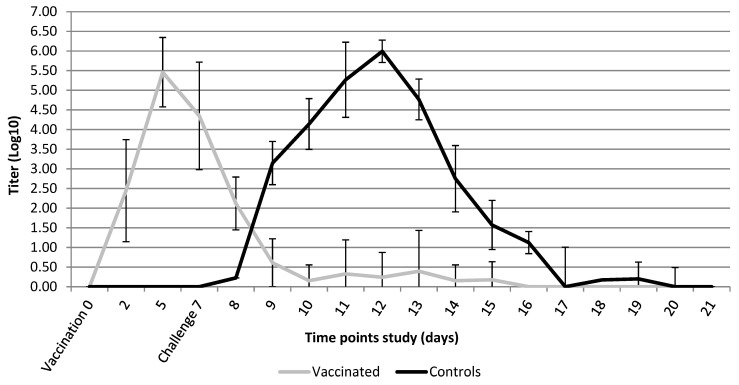
BPIV3 nasal shedding (mean titer log_10_ TCID_50_/mL) post-vaccination and 14 days post-challenge period. Shedding in the vaccinated group before challenge moment is caused by BPIV3 vaccine strain.

All nonvaccinated-challenged control animals were positive for BPIV3 nasal shedding post-challenge. The peak in nasal shedding was on day 5 post-challenge (Figure 1). The vaccinated animals still demonstrated low levels of shedding of the BPIV3 vaccine strain on the day of challenge, which dropped below detection limit on day 2 post-challenge. Challenge virus was detected in the nasal secretions of only one of the vaccinated animals on one day (day 5), while in all other vaccinated animals BPIV3 infection was cleared.

**Figure 2:**
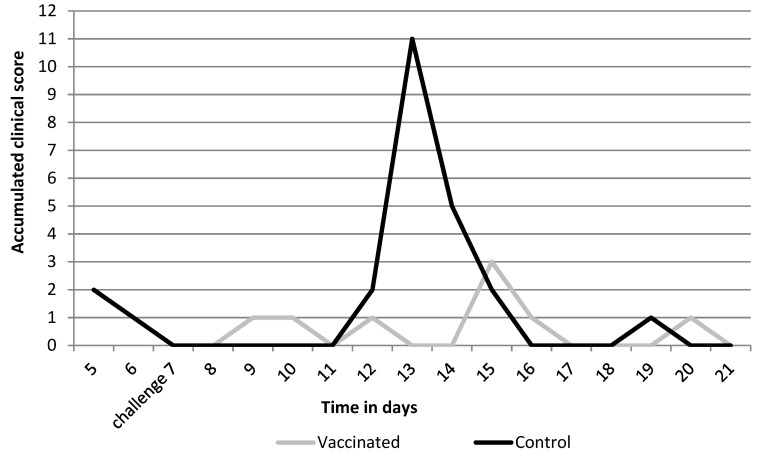
Sum of clinical scores per group after challenge.

Clinical signs (Figure 2) were seen in the control group and consisted of nasal discharge, coughing, and increased respiration rate. Based on the sum of scores over the 14-day time period, no statistically significant differences were seen for clinical signs (*p* = 0.3462), however, during the peak of clinical signs on day 6 and 7, a statistically significant difference was seen where control animals had higher scores than vaccinated animals (day 6 *p* = 0.03 and day 7 *p* = 0.03).

### 3.2. Changes in Ct Values of Genes after Vaccination, or Challenge, or Vaccination and Challenge

In order to reduce complexity of results, not all time points are presented here. For the vaccinated group, Ct values on day of vaccination (day 0, just before vaccination) were used as a reference point, and fold-change was calculated for day 5 (two days before challenge) and between day 7 (just before challenge, reference point) and day 12 (five days after challenge). For the nonvaccinated-challenged group, day of challenge day 7 (just before challenge) was taken as a reference point, and fold-change was calculated for day 12 (five days after challenge). Several analyses were done to justify the comparisons of the two groups, using values of samples collected at different time intervals and from animals at different ages. For genes that showed a significant change in expression, the first comparison of the Ct values of the two groups was performed on day 0. There were no significant differences in expression levels of those genes (average values in vaccinated animals were only 1.26-fold higher than in the control animals). Expression levels for CXCL10 and IFNG were 4.8 and 2.6 times higher, respectively, in the vaccinated animals, most likely due to two animals with mild symptoms of non-respiratory disease in the vaccinated group. The differences were not significant (Student’s *t*-test, two-sample equal variance), but they may have led to an underestimation of the fold-increase in the vaccinated animals for these two genes. The next comparison was of the values (R = ratio) between day 0 in the vaccinated animals and day 7 in the control animals; on average values were lower in the vaccinated animals (0.56-fold lower). The differences were significant for the following genes: FAS (R = 0.6, *p* = 0.01) and TLR4 (R = 0.6, *p* = 0.01), both caused by a low SD; and IRF7 (R = 06, *p* = 0.02), LOC512672 (R = 0.5, *p* = 0.03), CCL5 (R = 0.2, *p* = 0.04), IL18 (R = 0.3, *p* = 0.00), and LYZ (R = 0.3, *p* = 0.03), all five caused by an increase in controls on day 7. Therefore, the indicated fold-increase of these genes in control animals is an underestimation.

The focus of this investigation is on the difference in gene expression levels between vaccinated- and nonvaccinated-challenged animals. Therefore, only a summary of the results is given in this manuscript, which will be further evaluated in the discussion.

**Figure 3:**
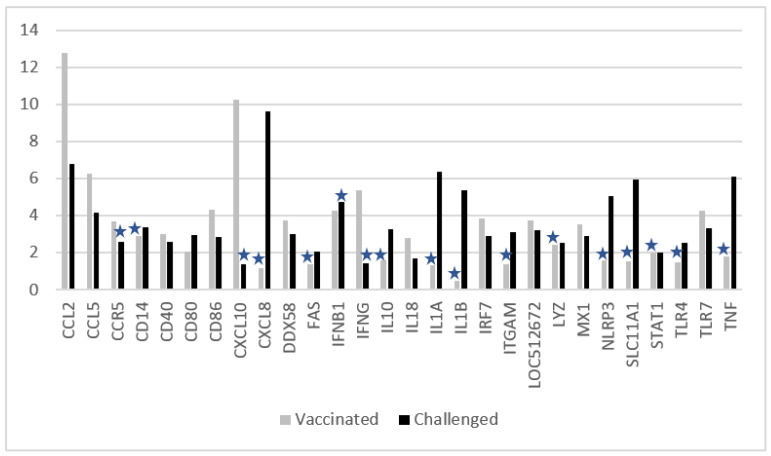
Comparison of gene expression (fold-change on *Y*-axis) of vaccinated- versus nonvaccinated-challenged animals. Genes are shown which had significant changes in Ct values five days after vaccination or five days after challenge (see materials and methods). Asterisks indicate non-significant changes according to the arbitrary criteria described in the materials and methods section.

In Figure 3 the results are shown for the genes that had significant changes in the expression between the two time points: (i) for the vaccinated group before vaccination and five days later, and (ii) for the nonvaccinated-challenged group before challenge and five days later.

In the vaccinated group, five days after vaccination CCL2, CCL5, CCR5, CD40, CD80, CD86, CXCL10, DDX58, IFNB1, IFNGG, IL18, IRF7, LOC512672, MX1, and TLR7 levels were significantly increased.

In the nonvaccinated-challenged control group, on day 5 after challenge CCL2, CCL5, CD14, CD40, CD80, CD86, CXCL8, DDX58, FAS, IL1A, IL1B, IL10, IRF7, ITGAM, LOC512672, LYZ, MX1, NLRP3, SLC11A1, STAT1, TLR4, TLR7, and TNFα were significantly enhanced in comparison to just before challenge.

In comparison to the nonvaccinated-challenged animals, the vaccinated animals had significantly higher induction of CCR5, CXCL10, IFNB1, IFNGG, and IL18 genes.

On the contrary, in comparison to the vaccinated animals, the nonvaccinated-challenged animals had significantly higher induction of CD14, CXCL8, FASLG, IL1A, IL1B, IL10, ITGAM, LYZ, NLRP3, SLC11A1, STAT1, TLR4, and TNF.

Both vaccinated and challenged animals had increased levels of CCL2, CCL5, CD40, CD80, CD86, DDX58, LOC512672, IRF7, MX1, and TLR7.

**Figure 4:**
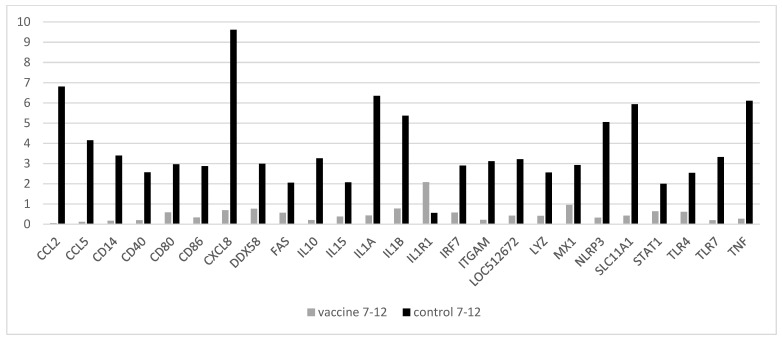
Comparison of gene expression (fold-change on *Y*-axis) of non-vaccinated control animals versus vaccinated animals after challenge with BPIV3 from day 7 to 12. All genes are shown which had significant changes to Ct values five days after challenge in both groups (see materials and methods). Only IL1R1 was significantly increased in the vaccinated group.

Figure 4 shows the overall Ct fold-changes of genes that showed a significant increase in both groups five days after challenge (day 12) in comparison to day 7. Interestingly, five days after challenge the values in the group that were vaccinated and challenged were almost all back to normal levels or lower as measured before vaccination, except IL1R1, which showed a 2.1-fold increase.

## 4. Discussion

The results of this study describe the immediate immune response five days after intranasal vaccination with a bivalent live viral BRD vaccine in young calves, based on levels of mRNA molecules of 84 target genes in nasopharyngeal mucosal samples. The results of vaccination were compared to responses after BPIV3 challenge.

This is the first report describing the difference in immune gene expression in the nasal mucosae induced by intranasal vaccination with a bivalent live BRSV-BPI3V vaccine formulation in comparison to wild-type infection with a virulent BPI3V strain. In most other similar in vivo studies [16,17], the focus was on BRSV infections because there is currently no vaccine for human RSV, and therefore BRSV is used as an alternative animal model [1].

Since the measurement of the mucosal immune gene expression was not the primary purpose of this animal study, some aspects of it’s design were not optimal, but we have demonstrated that the overall results give a clear impression of the innate response.

Firstly, it should be noted that the vaccine response is induced by two live, attenuated viruses, given with a syringe in the nasal cavity, while the challenge was only one, virulent BPIV3 strain, administered as an aerosol. Although these differences might have an impact on the reactivity of cells and tissues of the immune system, and therefore on the Ct values measured in this study, the data represent a real-life situation and can serve as a basis for further detailed research. Moreover, BPIV3 causes mild upper respiratory tract symptoms [18] such as nasal discharge and coughing, indicating that the nasopharyngeal mucosa is a target tissue for BPIV3 infection, as is the case for the vaccine viruses.

Secondly, although changing mRNA levels were not confirmed by functional assays for the respective immune-modulating molecules, this clearly gives a picture of the response of the tissues in the upper respiratory tract to the administered viruses, vaccine, or challenge strains. Also, post-translational activation was not detected using this RT-qPCR method. In addition, for most of these molecules the actual activity and function has not been proven in the bovine immune system but was extrapolated from murine or human data and similarity of the amino acid sequences. Highly sensitive assays would be required to assess the actual biological activity of these proteins and the increase in expression levels in animal samples.

Thirdly, the composition of the samples was not investigated further since sampling had to be performed in such a way that the RNA was preserved and fixed. Consequently, the number or ratio of different cell types or other mucosal material was not known and could influence RNA levels for different target genes. For example, the microbiome can have an impact on the status of the local mucosal immune system [19]. Since the sample collection was performed in a standard manner and Ct values were normalized by the ΔΔCt method using five house-keeping genes, it is plausible to assume that there are no big differences, which was confirmed by the relatively low variation of values between animals within the same group. In addition, the possible effect of sampling on the expression of the genes could have affected the results. However, samples were all collected and processed in the same standard manner with an unavoidable, intrinsic variability.

There could have been some effect on gene expression caused by underlying subclinical disease or conditions unrelated to vaccination or challenge, which is an inevitable potential complication when performing studies with very young colostrum-deprived calves. The data were checked for extreme values in certain animals and at specific time points but were absent or had no major impact on the conclusions.

It is interesting to observe that this live, intranasal vaccine can induce (almost complete) protection against nasal shedding of BPIV3 and thus inhibition of viral replication in the nasal mucosae, as well as against clinical symptoms of BRD within seven days after vaccination in very young calves. The protection seen can occur due to several mechanisms. Firstly, by induction of the non-specific, innate immune response, described by Wheat et al. [20] using intranasal administration of liposome-TLR complexes. This mechanism is also known as trained innate immunity, which has memory-like traits and could be boosted by challenge with BPIV3. Secondly, an antigen-specific, adaptive immune response may also have been induced, probably consisting of cellular-mediated activity, since at challenge no neutralizing antibodies were present in the sera of the vaccinated animals.

IgA and IgM were not measured in nasal fluid, but they are not expected to be present seven days after vaccination [21,22], especially in mucosae. Therefore, a strong non-specific innate immune response supported by low-level antigen-specific cellular response may result in protection. We know from other studies that protection is induced for the BRSV component of this vaccine after only 5 days [11].

The activity of γδT-cells, Natural Killer (NK) T-cells [23], or other specific T-cells was not determined in this study, but since γδT-cells are abundantly present in bovine mucosa, they are expected to play an important role in the early onset of this protective mechanism as well as the activity of NK T-cells, monocytes, and macrophages with respect to trained, innate immunity [10,24]. Further investigations, using techniques such as cell sorting based on specific markers and (single cell) RNAseq analysis, to determine the presence and activity of different cell types in nasal secretion after vaccination or challenge could indicate their potential contribution to this mechanism.

In this study we found that CCR5, CXCL10, IFNβ1, IFNγ, and IL18 were upregulated in the vaccinated group and not in the nonvaccinated-challenged group. CD14, CXCL8, FAS, IL10, IL1A, IL1B, ITGAM, LYZ, NLRP3, SLC11A1, STAT1, TLR4, and TNFα were upregulated in the nonvaccinated-challenged animals but not in the vaccinated animals. This indicated that the immune pathways were stimulated differently after vaccination with this bivalent formulation when compared to challenge with BPIV3, and that vaccination prevented the upregulation of (i) pro-inflammatory cytokines such as IL1A, IL1B, and TNFα, which are known to be involved in causing pathological pain and fever; (ii) NLRP3, FAS, or SLC11A1, involved in protection and damage of cells, like apoptosis and induction of IL1B; (iii) CXCL8 (IL-8), involved in inflammation by attracting neutrophils and other granulocytes [25]; (iv) ITGAM, an α_M_β_2_ integrin that supports inflammation by regulation of leukocytes; and (v) IL-10, which can inhibit IFNγ synthesis.

Vaccination leads to IFNγ induction, which is the most important cytokine for a cellular immune response, further confirmed by increased levels of (i) CCR5, a chemokine receptor expressed on Th1 cells which plays an important role in the CD8+ T cell early memory response [26]; (ii) CXCL10, that mediates immunity to respiratory syncytial virus infection by augmenting dendritic cell and CD8+ T cell efficacy [27]; and (iii) IL-18, a pro-inflammatory cytokine inducing IFNγ production.

In addition to the data set presented above, samples taken on the day of challenge (just before challenge) in the vaccinated animals were collected and analyzed. Along with those already mentioned to be elevated on day 5 after vaccination, genes such as CD4, CD8A, CXCR3, FASLG, IL10, and ITGAM were significantly increased, representing cellular immune defense mechanisms, memory, and anti-inflammatory activity of IL-10 in the nasal mucosae.

Antiviral pathways were induced in vaccinated- and nonvaccinated-challenged animals (IRF7 and MX1), but caused a more elevated downstream immune response in the vaccinated animals only, suggesting that wild-type viruses could interfere with processes in the antiviral pathway. BRSV and BPIV3 have previously been shown to block production and downstream signaling of Type I IFNs, and specifically the wild-type BPIV3 modulates signaling pathways downstream of the Type III IFN receptor to block production of several specific molecules that aid in a productive antiviral response [28]. In analogy, the non-structural proteins of BRSV were shown to block the induction of IFN-α/β gene expression and thereby evade the immune response and establish the virulent nature of wild-type BRSV [29]. We show here that vaccination with the two attenuated viruses allows an antiviral response.

Studies comparable to our investigation were performed by Tizioto [17] and Johnston et al. [16] in which older calves of two different breeds were infected with a wild-type BRSV strain, and RNAseq analyses were done on bronchial lymph node tissue. In both studies, pathways were identified, such as interferon signaling and pattern recognition receptors of bacteria and viruses, induction of pro-inflammatory chemokines and cytokines for the initiation of the innate antiviral immune response, and other genes were also identified in the vaccinated- and/or nonvaccinated-challenged animals in this study. However, the design of these experiments revealed no indication of the early onset of protection after vaccination, only the response of target tissue infected with wild-type viruses.

## 5. Conclusions

In conclusion, in comparison to the virulent wild-type field isolate, the two virus strains in the vaccine have lost their capacity to inhibit the immune response, resulting in the induction of an antiviral state followed by a very early onset of downstream innate and specific cellular immune pathways, leading to protection. The exact genomic changes of these vaccine strains leading to their attenuation have not been identified. These data represent a real-life situation and can serve as a basis for further detailed research.

## Figures and Tables

**Table 1:** Serological response in VN titers against BRSV and BPIV3.

Title: Log2 VN Titers		Time in Days			
		BRSV	BPIV3		
Animal	Group	0	0	7	21
1	Vaccinated	2.0	1.0	1.0	6.0
2	Vaccinated	1.0	1.0	1.0	7.0
3	Vaccinated	1.0	1.0	1.5	7.0
4	Vaccinated	1.0	1.0	1.0	9.0
5	Vaccinated	1.5	1.5	1.5	7.0
6	Vaccinated	1.0	1.0	2.0	7.0
	**Average**	**1.3**	**1.1**	**1.3**	**7.3**
7	Controls	1.0	1.0	1.0	7.0
8	Controls	1.0	1.0	1.0	3.5
9	Controls	1.0	1.0	1.0	5.5
10	Controls	1.0	1.0	1.0	6.0
11	Controls	1.0	1.0	1.0	3.0
12	Controls	1.0	1.0	1.0	4.5
13	Controls	1.0	1.0	1.0	5.0
	**Average**	**1.0**	**1.0**	**1.0**	**4.9**

BRSV, bovine respiratory syncytial virus; BPIV3, bovine parainfluenza type 3 virus.

## Data Availability

The data presented in this study are available on request from the corresponding author. The data are not publicly available due to regulatory reasons.

## Supplementary Materials

The following are available online at https://www.mdpi.com/article/10.3390/vaccines10010104/s1, File S1: Data presented in Figure 3 and Figure 4.
